# Controlled Ovarian Stimulation with recombinant-FSH plus recombinant-LH vs. human Menopausal Gonadotropin based on the number of retrieved oocytes: results from a routine clinical practice in a real-life population

**DOI:** 10.1186/s12958-015-0080-6

**Published:** 2015-07-25

**Authors:** Alberto Revelli, Grazia Pettinau, Gemma Basso, Andrea Carosso, Alessandro Ferrero, Cecilia Dallan, Stefano Canosa, Gianluca Gennarelli, Daniela Guidetti, Claudia Filippini, Chiara Benedetto

**Affiliations:** Gynecology and Obstetrics, Physiopathology of Reproduction and IVF Unit, Department of Surgical Sciences, University of Torino, S. Anna Hospital, Torino, Italy; LIVET Infertility and IVF Clinic, Torino, Italy; Statistics, Department of Surgical Sciences, University of Torino, MolinetteHospital, Torino, Italy

**Keywords:** Recombinant FSH, Recombinant LH, Human menopausal gonadotropin, In vitro fertilization, IVF outcome, Pregnancy rate

## Abstract

**Background:**

The association of recombinant FSH (rFSH) plus recombinant LH (rLH) is currently used for Controlled Ovarian Stimulation (COS) in human IVF, but its efficacy has, to date, not yet been compared to that of human Menopausal Gonadotropin (hMG), the FSH + LH activity-containing urinary drug.

**Methods:**

Eight hundred forty-eight (848) IVF patients classified as expected “poor” or “normal” responders according to antral follicle count (AFC) and basal (day 3) FSH were treated with rFSH + rLH (2:1 ratio, *n* = 398, Group A) or hMG (*n* = 450, Group B). Data were collected under real-life practice circumstances and the pregnancy rate with fresh embryos was calculated by stratifying patients according to the number of retrieved oocytes (1–2, 3–4, 5–6, 7–8, >8).

**Results:**

Overall, the pregnancy rate in both groups progressively improved according to the number of oocytes retrieved. When comparing patients within the same subgroup of oocyte yield, Group A and B showed a comparable outcome up to the reported highest yield (>8). When more than 8 oocytes were available, Group A had a significantly better pregnancy rate outcome. Patients’ characteristics did not significantly differ between the two groups and the better outcome in the best responding patients in Group A was confirmed by a multivariable logistic regression analysis, that showed that both the use of rFSH + rLH and the total number of retrieved oocytes increased the probability of pregnancy with odd ratio (OR) of 1.628 and 1.083, respectively.

**Conclusions:**

When comparing patients with the same number of retrieved oocytes under real-life circumstances, the association of rFSH + rLH results in a significantly higher pregnancy rate than hMG when more than 8 oocytes are retrieved. The reason(s) for this are unknown, but a more favorable effect on oocyte quality and/or endometrial receptivity could be involved.

## Background

To date, no confirmed evidence is yet available as to which gonadotropin (or gonadotropin association) is more effective when performing controlled ovarian stimulation (COS) in human in vitro fertilization (IVF). The heterogeneity of infertility condition and of patients’ profile, together with the availability of several medications that can be combined in various different regimens, lead to the need of individualizing COS regimen as a specific protocol could be effective on one definite patient and not in another [1].

Randomized Controlled Trials (RCTs) are recognized as the “gold standard” for assessing the efficacy of a given therapeutic regimen, but they cannot provide a true indication of effectiveness as they operate in an idealized environment and measure efficacy in limited, standardized populations. Moreover, both patients and physicians in a clinical trial may behave differently simply because they know to be in a trial and being observed (the so-called Hawthorne effect) [2]. As a consequence, the conclusions of RCTs are not always a useful aid for decision-making, as assessing the value of a drug (or of a treatment protocol) requires the comprehension of its impact on current management in a practical real-life setting [3]. Real-life data may be obtained from the retrospective evaluation of a database, and provide additional insights coming from a more realistic clinical environment; internationally, this additional effectiveness assessment is increasingly used in the development of evidence-based documentation of a treatment value, particularly when a medical treatment is submitted to evaluation for pricing and reimbursement decision [4].

In the last decades, a bulk of published data comparing human Menopausal Gonadotropin (hMG) vs recombinant Follicle Stimulating Hormone (rFSH) in human IVF have been published, but they have not been able to demonstrate any significant difference in live birth rate [5, 6], although showing a higher oocyte yield with the recombinant molecule [6, 7]. After recombinant Luteinizing Hormone (rLH) was made available, both retrospective [8, 9] and prospective [10–14] studies reported that rLH addition to rFSH was unable to increase the number of oocytes and/or improve the outcome vs. rFSH alone. Differently, however, other studies showed increased pregnancy and implantation rates in some subset of patients receiving rLH in addition to rFSH [15, 16], and a recent, large meta-analysis suggested that rLH supplementation might result in a higher clinical pregnancy rate in the overall population, and particularly in the poorly responding patients [17]. Rather surprisingly, no large studies comparing hMG with rFSH + rLH have been performed, and the available data are still scarce and inconclusive.

The aim of the present study was to compare, applying the real-life data approach on a routine clinical activity, the effectiveness of COS protocols with rFSH + rLH vs hMG in a well-defined subset of IVF patients. In order to exclude the interference of a different oocyte yield, we evaluated the pregnancy rate obtained after patients’ stratification in subgroups according to the number of retrieved oocytes.

## Methods

### Patients

The study was authorized by the local Ethical Committee and was registered at Clinical Trials.gov with number NCT02322398.

Data were collected from the clinical charts of our IVF Unit database, including all patients undergoing IVF in the period between 2010–2014, in which the Italian rules on assisted reproduction allowed the use of all mature oocytes and the freezing of surplus embryos. IVF patients were classified according to basal antral follicle count (AFC) and basal (day 3) FSH levels as “expected poor responders” (ePR; AFC ≤ 7, FSH ≥ 12 U/l) “expected normal responders” (eNR; AFC 8–15, FSH 8.1–11.9 U/l), or “expected high responders” (eHR; AFC ≥ 16, FSH ≤ 8 U/l). Among 3,416 cases recorded, approximately two thirds matched the criteria defined as ePR or eNR, and 848 of them received a COS with LH activity-containing medications, whereas the others were stimulated using FSH alone. In detail, 398 patients (Group A) were stimulated with rFSH + rLH, whereas 450 patients (Group B) were treated with hMG. The clinical characteristics of enrolled patients appear in Table 1.

**Table 1:** Anthropometric and clinical data of all patients. Women in Group A received r-FSH + r-LH/2:1, those in Group B  hMG. Data are expressed as mean ± SD or as percentage

	Group A	Group B	p
Number of patients	398	450	
Age (yrs)	36.7 ± 4.0	36.8 ± 4.1	ns
BMI	22.6 ± 3.6	22.0 ± 3.1	ns
Basal (day 3) FSH (IU/l)	8.1 ± 3.3	8.1 ± 3.2	ns
Antral Follicle Count (AFC)	9.9 ± 6.2	10.3 ± 6.7	ns
Main infertility cause (%)			ns
*Anovulation*	1	2	
*Endometriosis*	11	8	
*Male*	44	51	
*Tubal*	16	13	
*Unexplained*	17	17	
*Mixed*	11	9	
Smoke habit (%)	16.3	20.2	ns
Total Gonadotropin dose (IU)	2705 ± 1598	2837 ± 1171	ns
Peak E2 (pg/ml)	2644 ± 1117	2303 ± 990	ns
Retrieved oocytes/patient	6.7 ± 4.2	6.1 ± 4.1	ns
MII oocytes/patient	4.3 ± 2.7	4.2 ± 2.3	ns
Embryo morphological score	6.1 ± 3.6	6.2 ± 3.6	ns
Top scored (>8/10) embryos (%)	21	20	ns
Number of transferred embryos	2.0 ± 0.1	2.0 ± 0.1	ns
Endometrial thickness (mm)	10.8 ± 2.3	10.6 ± 2.2	ns

### IVF treatment protocols

Patients in Group A (*n* = 398) received either a starting dose of 150–300 IU/d recombinant FSH (rFSH; Gonal F®; Merck-Serono, Germany) plus 75–150 IU/d recombinant LH (rLH; Luveris®; Merck-Serono, Germany) in 2:1 ratio, or 150–300 IU/d rFSH + rLH/2:1 (Pergoveris®, Merck-Serono, Germany). On day 6–7 of ovarian stimulation, the gonadotropin dose was adapted according to the ovarian response, always maintaining a rFSH:rLH/2:1 ratio. Patients in Group B (*n* = 450) received 150–300 IU/d hMG (Meropur®, Ferring Pharmaceuticals, Germany or Merional®, IBSA, Switzerland). On day 6–7 of ovarian stimulation, the hMG dose was eventually adjusted according to the ovarian response.

Both medications were administered within a “long” protocol with GnRH-agonists or a “short” protocol with GnRH-antagonists. In the absence of any pre-fixed criteria, the COS regimen (type of protocol and type of medication) was decided and prescribed by different physicians of the Unit according to their own clinical experience, as per real-life clinical practice. As a common background, the choice of the starting gonadotropin dose was based on age, body mass index (BMI), AFC, basal FSH as well as on the response to previous COS.

The classical “long” protocol was performed administering the GnRH-agonist buserelin (Suprefact®, Hoechst, Germany; 900 mcg/d intranasally) from day 21 of the incoming cycle. After approximately two weeks, pituitary suppression was verified (appearance of a menstrual bleeding, serum estradiol <50 pg/ml, endometrial thickness <3 mm) before starting COS. In the “short” protocol, the GnRH-antagonist cetrorelix (Cetrotide®, Merck-Serono, Germany) was started at a subcutaneous dose of 0.25 mg/d according to a flexible schedule, when at least one follicle ≥14 mm diameter was observed at ultrasound (US).

### IVF cycle management

COS was monitored by serial transvaginal US plus serum estradiol (E2) measurements performed every second day from stimulation day 6–7. The cycle was cancelled when no more than one follicle ≥11 mm diameter was seen at US and serum E2 was <80 pg/ml the day of the first checkpoint. From stimulation day 6–7 onward, COS continued until at least one dominant follicle reached 18 mm diameter, with appropriate E2 levels. At this point, ovulation was triggered by injecting 10,000 IU of hCG (Gonasi HP®, IBSA, Switzerland) subcutaneously, and transvaginal US-guided oocyte aspiration (OPU) was performed approximately 36–37 h later under local anesthesia (paracervical block). Classical IVF or ICSI were performed according to clinical indication. After two days of in vitro culture, embryos were scored according to Holte [18] and 1–3 embryos were transferred in utero using a soft catheter (Sydney, Cook, Australia) under US guidance. If several good scoring embryos were obtained, surplus embryos were frozen and kept in liquid nitrogen for further use. The luteal phase was supported administering 180 mg/d natural progesterone (Crinone 8®, Merck-Serono, Germany) for 15 days. Pregnancy was assessed by serum hCG assay after 15 days from embryo transfer (ET) and then confirmed if at least one gestational sac was visualized at transvaginal US after two further weeks. Only cases with US confirmation of pregnancy were counted in the calculation of pregnancy rates, whereas biochemical pregnancies were not considered.

### Statistical analysis

The primary end point of the study was to evaluate the pregnancy rate per embryo transfer (PR/ET) with fresh embryos according to the number of retrieved oocytes and to the type of gonadotropin used (recombinant vs. urinary). Actually, the following subgroups were evaluated: 1–2, 3–4, 5–6, 7–8, >8 retrieved oocytes; in each subgroup the PR/ET obtained using rFSH + rLH or hMG were then compared. The secondary endpoint was to evaluate the influence of basal day 3 FSH levels, maternal age and type of pituitary down-regulation on the primary endpoint. For this reason, results were also stratified by characteristics as basal circulating FSH level (<10 or ≥ 10 U/l), age at the time of IVF start (<38 years or ≥38 years) and adoption of either “long” or “short” protocol for pituitary down-regulation.

Descriptive data were expressed either as absolute values, percentages or means as appropriate. Comparisons between groups for continuous variables were performed using the two-tailed *t*-test for unpaired data. The Chi-square test was used to compare the differences in PR/ET per number of oocytes in the two Groups of patients. Multivariate logistic regression analysis was performed to further evaluate the impact of the type of medication on the probability of obtaining a pregnancy: the regression model used age, BMI, smoke habit, and number of retrieved oocytes as covariates. Significance was defined as a p value <0.05.

## Results

Groups A and B did not significantly differ from any of the recorded anthropomorphic variables describing patients’ population, suggesting the absence of a selection bias in the allocation of patients in the two groups (Table 1). Similarly, the recorded variables concerning COS were similar in both groups, suggesting a homogeneous cycle management regardless of which medication was used (Table 1).

**Fig. 1:**
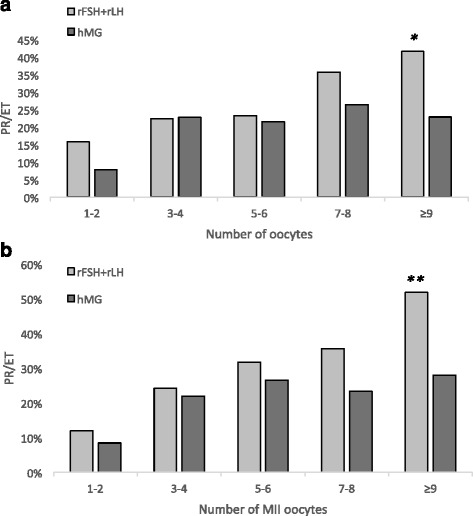
Pregnancy rate per embryo transfer (PR/ET) in subgroups of patients stratified according to the number retrieved oocytes (**a**) or to the number of mature (MII) oocytes (**b**). Pale columns correspond to patients who received rFSH + rLH/2:1 (Group A, *n* = 398), dark columns to patients who received hMG (Group B, *n* = 450). *p = 0.0038; **p = 0.013

The overall trend of the clinical PR/ET in the two study groups, stratified according to the number of retrieved oocytes, is summarized in Fig. 1a: as a general trend, IVF outcome showed a progressive improvement in parallel with the increase in the number of retrieved oocytes in both Groups.

Comparing subgroups of patients who had the same oocyte yield (1–2, 3–4, 5–6, 7–8, or > 8 oocytes retrieved at OPU), the difference between Group A and B became progressively more pronounced in parallel with the increasing number of available oocytes (Fig. 1a). Indeed, the clinical PR/ET of Groups A and B were similar up to 6 retrieved oocytes; with 7–8 oocytes, a non-significant trend toward better results was observed in Group A; with more than 8 oocytes available, Group A obtained a significantly higher success rate (p = 0.038; Fig. 1a). When comparing the clinical, hormonal and US parameters of patients who obtained more than 8 oocytes in the two Groups (Table 2), no significant difference was noticed, suggesting once again that the better IVF outcome in Group A was not due to a patients’ selection bias. The better performance in Group A was even more pronounced when only mature (MII) oocytes were considered: with more than 8 MII oocytes, the PR/ET in Group A was significantly higher than in Group B (p = 0.013, Fig. 1b). Interestingly enough, both in the whole group (Table 1) and in the subgroup of the most responsive patients (Table 2), no significant difference in the embryo morphological score, the proportion of top-scored embryos, and the endometrial thickness was found, suggesting that subtle factors, not detectable by morphological methods, could be at the basis of the observed difference in the clinical PR. The multivariable logistic regression model confirmed that both the use of rFSH + rLH and the total number of retrieved oocytes increased the probability of pregnancy with OR of 1.628 (C.I. 1.163–2.279) and 1.083 (C.I. 1.042–1.126), respectively (Table 3).

**Table 2:** Anthropometric and clinical data of patients obtaining more than 8 oocytes at ovum pick-up. Women in Group A received r-FSH + r-LH/2:1, those in Group B hMG. Data are expressed as mean ± SD or as percentage

	Group A	Group B	p
Number of patients	101	109	
Age (yrs)	36.2 ± 4.3	35.7 ± 4.5	ns
Basal (day 3) FSH (IU/l)	7.1 ± 2.5	7.2 ± 2.8	ns
BMI	22.7 ± 3.9	22.3 ± 2.9	ns
Main infertility cause (%)			ns
*Anovulation*	0	0.9 %	
*Endometriosis*	8.9	8.3	
*Male*	43.6	43.1	
*Tubal*	11.9	18.3	
*Unexplained*	24.7	23.8	
*Mixed*	10.9	5.5	
Smokehabit (%)	14.8	20.2	ns
Total Gonadotropin dose (IU)	2453 ± 1539	2801 ± 1117	ns
Peak E2 (pg/ml)	2924 ± 1175	2649 ± 964	ns
Retrieved oocytes/patient	12.4 ± 3.6	11.9 ± 3.3	ns
MII oocytes/patient	9.2 ± 2.4	9.1 ± 2.3	ns
Embryo morphological score	7.1 ± 2.8	6.9 ± 3.0	ns
Top scored (>8/10) embryos (%)	28	25	ns
Number of transferred embryos	2.3 ± 0.7	2.4 ± 0.7	ns
Endometrial thickness (mm)	10.6 ± 2.5	10.8 ± 2.2	ns

**Table 3:** Odd ratio estimates according to the multivariable logistic regression analysis. The analysis was used to test the impact of the type of medication used in COS (rFSH + rLH or hMG) on the likelihood of obtaining a pregnancy, using age, BMI, smoke habit and number of retrieved oocytes as covariates. Variables with OR > 1 and 95 % confidence limits both above 1 significantly affect the final outcome (pregnancy)

	Odd ratio	95 % confidence limits	
Age	0.901	0.863	0.940
BMI	0.978	0.929	1.029
Smoke habit	0.944	0.564	1.581
Type of medication used in COS	1.628	1.163	2.279
Number of retrieved oocytes	1.083	1.042	1.126

Stratification of the sample according to some baseline patients’ characteristics showed that the better IVF outcome observed in Group A for the most responsive patients was independent from the basal FSH level (this was observed both when it was >10 U/l and when it was <10 U/l), from age (it was observed both for women below 38 years and for those above), and from the type of down-regulation used (it was observed both in the long and short protocols) (not shown).

## Discussion

Even if FSH alone may be sufficient to obtain follicular growth in COS, endogenous LH may be so deeply suppressed by pituitary down-regulation that some LH activity may be useful to achieve adequate steroidogenesis and develop the capacity of the follicle to ovulate and luteinize when exposed to hCG [19, 20]. Before recombinant-LH availability on the market, highly purified hMG was the only source of exogenous LH activity; hMG, however, contains a little amount of LH, most of its LH activity deriving from human chorionic gonadotropin (hCG) content rather than from LH itself [21]. Indeed LH and hCG share more than 80 % sequence homology and bind the same receptor, the luteinizing hormone-chorionic gonadotropin receptor (LHCGR) [22, 23]. However, in vitro studies showed that LHCGR is able to differentiate the LH and hCG action at the molecular level [24], reflecting the different role of the two molecules in human physiology: during follicle development and first trimester pregnancy, respectively.

Binding of the ligand to LHCGR activates several intracellular signaling pathways: i) the cyclic AMP-protein kinase A (cAMP/PKA) pathway, which stimulates steroidogenesis [25] and apoptosis [26] in granulosa cells; ii) the AKT-pathway, involved in protection from apoptosis [24], and iii) the ERK1/2-pathway, involved in resumption of oocyte meiosis and in proliferation, differentiation and survival of granulosa cells [27]. So far, these pathways play a crucial role in the final stages of maturation of human oocytes and follicles. Cyclic AMP-protein kinase A (cAMP/PKA) pathway is more rapidly activated after LH than after hCG exposure, but the effect of hCG is more persistent, due to its longer half-life (60–120 min for LH vs. several hours for hCG) [28, 29] generating similar steroidogenetic activity. In contrast, AKT- and ERK1/2-pathways are significantly different after LH o rhCG exposure, showing a more favorable effect of LH as anti-apoptotic and oocyte maturation modulator [24]. The long half-life of hCG, moreover, is able to exert a down-regulation and internalization of the LHCGR, that was described in cultured LH-sensitive cells after repeated, low dose hCG administration [30], and in the long run reduces the sensitivity of the receptor in the target tissues.

To date, very limited clinical data have been generated comparing the LH activity of rLH vs. that of the hCG contained in hMG in patients undergoing IVF. Hormonal profiles in serum and follicular fluid obtained using the association rFSH + rLH during COS were found to be similar to those obtained with hMG, showing that their respective steroidogenetic activity at the follicular level was comparable [31]. In a randomized prospective study including a small number of FSH-stimulated patients, normo-gonadotropic women older than 35 obtained a higher number of preovulatory follicles and of mature oocytes if supplemented with rLH vs. hMG [32]. Another small, prospective pilot study on 122 patients undergoing IVF reported comparable outcome with rFSH + rLH vs. hMG in terms of embryo quality, pregnancy rate (PR), and implantation rate (IR) [33]. Results from the German IVF Registry, including more than 4,000 cycles, showed that oocyte yield, PR and IR were significantly higher in patients treated with the combination of rFSH + rLH compared to women treated either with rFSH + hMG or hMG alone [34].

Some recent studies, including a huge retrospective analysis on 400,135 IVF cycles [35] indicate that IVF outcome improves in parallel with increasing oocyte yield and the best chance of live birth with fresh embryo transfer is obtained with a number of retrieved eggs around 15 [35–37]. It is also well known that recombinant gonadotropins are able to induce the retrieval of more oocytes than hMG [6, 7], but none of the studies showing a relationship between IVF outcome and oocyte yield considered which medication was used for COS.

In the present study, we applied an original approach comparing the clinical effectiveness of recombinant gonadotropins vs. hMG, stratifying a rather large number of IVF patients with homogeneous characteristics according to the number of retrieved oocytes. As both treatments under investigation were able to generate a similar number of oocytes, we were able to compare even subgroups having the same oocyte yield (1–2, 3–4, 5–6, 7–8, and more than 8), but treated with different medications. This is the first study, to our knowledge, that uses this approach to compare different COS regimens in IVF patients.

Our observation confirmed the importance of oocyte yield for a successful IVF, as a positive trend toward higher PR was observed in parallel with the number of retrieved oocytes and of mature oocytes. Also, the multivariable logistic regression analysis identified the number of retrieved oocytes as a variable significantly and independently affecting the probability of pregnancy (OR 1.083). It was observed, however, that the type of medication used for COS played a role in determining IVF outcome: in fact, a similar success rate with recombinant gonadotropins or hMG was achieved when the number of available oocytes was 1–6, a non-significantly higher pregnancy rate using rFSH + rLH with 7–8 oocytes and finally a significantly higher pregnancy rate with recombinant gonadotropins when more than 8 oocytes were retrieved at OPU. This finding was not justified by any clinical differences between the two groups, but depended, apparently, on the type of gonadotropin used. Also, the multivariable logistic regression analysis showed that the type of medication used in COS significantly and independently affected the probability of pregnancy, with a highly significant OR of 1.628 in favor of recombinant gonadotropins. Interestingly enough, it was recently reported that recombinant gonadotropins lead to a better IVF outcome than hMG in patients having an AFC above six [38].

The reason(s) why recombinant gonadotropins may be able to lead to a better IVF outcome than hMG in patients with good ovarian responsiveness, despite an even number of oocytes, is difficult to determine. As a matter of fact, when comparing Group A to Group B no difference in the morphology of the oocytes and of the derived embryos, nor in endometrial thickness was observed; we can thus speculate that the observed results could be due to subtle factors, not detectable when studying embryo morphology or endometrial US appearance. The embryo morphological score that we used [18] was proven to be quite precisely related to embryo implantation potential, but still it could be inappropriate to detect subtle differences in embryo competence, in turn possibly derived from oocyte quality. We performed embryo transfer on day 2 or 3, so unfortunately the blastocyst development rate and the blastocyst morphology could not be estimated in our patients. As for endometrial quality, the gene and protein expression in endometrial cells was reported to differ in hMG-treated and rLH-treated subjects [39, 40], and a precocious or prolonged hCG exposure (as when hMG is used) was observed to worsen endometrial receptivity in baboons [41], possibly for the down-regulatory effect of hCG on the LHCGR [30, 40]. Specifically, the existence of a 2-d delay in the activation/repression of two clusters of genes in endometrial cells was demonstrated, respectively, on day hCG + 7 vs. LH + 7; this different gene regulation could potentially affect the window of implantation in the endometrium [42], as LHCGR expression affects uterine receptivity [43]. Further, hCG was also reported to induce pro-apoptotic molecules in endometrial cells [44], and it was shown that the outcome of thawed embryo transfer in spontaneous cycles was better with endogenous LH surge than after a single hCG bolus [45].

In conclusion, we performed herein an original evaluation of the real-life data generated from routine clinical activity in order to assess the efficacy of different medications for COS. Studying several hundreds of IVF patients with homogeneous characteristics and classified as poor or normal responders to gonadotropins, we observed that hMG and the rFSH + rLH/2:1 association were similarly effective in generating a clinical pregnancy when less than 8 oocytes were retrieved, but recombinant gonadotropins were significantly more effective when 8 or more oocytes were available. These findings cannot be explained by a selection bias, by a difference in patients’ characteristics or by an uneven number of retrieved oocytes; they are likely linked to the type of gonadotropin used in COS, and depend on some subtle, different effect of LH and hCG on oocyte and/or endometrial characteristics. The more favorable clinical outcome we observed using recombinant gonadotropins in normal responders, in spite of a similar oocyte yield, could be further investigated in a large, prospective clinical trial; in parallel, a targeted basic research could be performed in order to clarify the subtle mechanisms of action of rLH and hCG at the follicular and endometrial level.
